# The Better Survival of MSI Subtype Is Associated With the Oxidative Stress Related Pathways in Gastric Cancer

**DOI:** 10.3389/fonc.2020.01269

**Published:** 2020-07-28

**Authors:** Lei Cai, Yeqi Sun, Kezhou Wang, Wenbin Guan, Juanqing Yue, Junlei Li, Ruifen Wang, Lifeng Wang

**Affiliations:** Department of Pathology, Xinhua Hospital, School of Medicine, Shanghai Jiao Tong University, Shanghai, China

**Keywords:** gastric cancer, microsatellite instability, Epstein-Barr virus, prognosis, bioinformatics analysis

## Abstract

**Background:** Gastric cancer (GC) is the third leading fatal cancer in the world and its incidence ranked second among all malignant tumors in China. The molecular classification of GC, proposed by the The Cancer Genome Atlas (TCGA), was added to the updated edition (2019) of WHO classification for digestive system tumor. Although MSI and EBV subtypes appeared as ever-increasingly significant roles in immune checkpoint inhibitor therapy, the underlying mechanisms are still unclear.

**Methods:** We systematically summarized the relationship between EBV, d-MMR/MSI-H subtypes and clinicopathological parameters in 271 GC cases. Furthermore, GSE62254/ACRG and TCGA-STAD datasets, originated from Gene Expression Omnibus (GEO) and TCGA respectively, were analyzed to figure out the prognosis related molecular characteristics by bioinformatics methods.

**Results:** Patients with MSI subtype had better prognosis than the MSS subtype (*P* = 0.013) and considered as an independent biomarker by the univariate analysis (*P* = 0.017) and multivariate analysis (*P* = 0.050). While there was no significant difference between EBV positive and negative tissues (*P* = 0.533). The positive prognostic value conferred by MSI in different cohorts was revalidated via the clinical analysis of GSE62254/ACRG and TCGA-STAD datasets regardless of race. Then key gene module that tightly associated with better status and longer OS time for MSI cases was obtained from weighted gene co-expression network analysis(WGCNA). *NUBP2* and *ENDOG* were screened from the gene cluster and oxidative phosphorylation, reactive oxygen species(ROS) and glutathione metabolism were analyzed to be the differential pathways in their highly expressed groups.

**Conclusions:** Our results manifested the significant prognostic value of MSI in Chinese GC cohort and comparisons with other populations. More opportunities to induce apoptosis of cancer cells, led by the unbalance between antioxidant system and ROS accumulation, lay foundations for unveiling the better prognosis in MSI phenotype through the bioinformatics analysis.

## Introduction

Gastric cancer (GC), a highly heterogeneous disease, is the third most common cause of cancer-related death worldwide with a particular high incidence and mortality in Asia (1). Although the operation, chemotherapy and radiotherapy were widely used, the therapeutical efficacy was still such limited for some patients. The advent and development of next-generation sequencing (NGS) has revolutionized our understanding of its pathogenesis and molecular alterations. TCGA had presented four distinct subtypes-Epsterin-Barr virus (EBV), microsatellite instability (MSI), chromosomal instability(CIN) and genome stable(GS) through comprehensive molecular evaluation of 295 primary gastric cancer (2–4). Recognition of molecular subtypes can indeed help to establish a new paradigm of cancer therapeutics especially as the development of immunotherapy. Nevertheless, each molecular subtype had divergent response and therapeutical effects to immunotherapy. Impressive results from some clinical trials have demonstrated that solid tumors with MSI phenotype had more significant responses to anti-PD1 inhibitors than that with Microsatellite Stable (MSS) in patients who failed conventional therapy and GC was one of them (5–7). Compared with GS and CIN, metastatic GC patients with the MSI and EBV subtype manifested a dramatic response to PD1 inhibitor (8). Furthermore, GC patients with MSS status could benefit from 5-FU-based adjuvant chemotherapy in TNM stage II–III (9). Therefore, correct evolution of EBV infection and MSI status could be served as a potential biomarker for anti-PD1/PD-L1 targeted therapy and 5-FU based traditional chemotherapy in GC.

High-Microsatellite Instability (MSI-H) phenotype has been widely acknowledged to be the predictive factor for immunotherapy as its high PD-L1 expression. Some researchers has represented that MSI is an independent predictive factor while others observed that there are no significant difference of prognosis between divergent MSI status (10–16). The complex interactions that involved in the p53 signal pathways or E2F/DP1 transcription factors may largely contribute to the outcome (17–19). It also has been revealed that the EBV infection may be connected with the GC carcinogenesis at an early stage though the exact mechanism is still unclear. Enhanced understanding of the clinicopathological and prognostic implications of these molecular subtypes will assist to acquire the reasonable evaluation of the biological behavior of tumors. In fact, considerable literatures have investigated the relations between MSI phenotype and their prognosis but the conclusion is still in the air (15). So did the similar condition in EBV subtype. For example, Ahn et al. and Setia et al. separately revealed a significant survival advantage for EBV associated gastric cancer (EBVaGC), whereas Genitsch et al. showed that there was no association between EBV infection and clinical outcome of GC patients (10, 20, 21).

Moreover, results from Shen et al. manifested that EBV+ patients had a poorer OS than EBV- patients (12). The discrepancies may be due to a number of factors, such as different ethnic background of the enrolled patients or multiple methods for detecting the presence of EBV/MSI alteration. Hence, more robust tools for detection of EBV/MSI phenotype and better-tailored investigation should be applied to elucidate the real contributions of them to prognosis in various regions.

Since most aforementioned data about MSI and EBV (+) GC are derived from studies of western population, little investigations have reported for Chinese cohorts. In this study, we adopted the most widely used methods to identify EBV infection (EBV–encoded RNA by *in situ* hybridization) and Mismatch Repair deficiency/High-Microsatellite Instability (d-MMR/MSI-H) status (joint application of immunohistochemical staining & PCR-based MSI testing according to NCI panel) in 279 Chinese GC patients. Then, the clinicopathological characteristics and prognostic significance of EBV+ and MSI were in-depthly explored in present study. In addition, the data derived from TCGA and GEO had been used to compare the clinical differences among diverse cohorts. The associated molecular mechanisms were also analyzed by utilization of the bioinformatics.

## Materials and Methods

### Patients and Samples

A total of 279 consecutive cases with gastric cancer were included at our institution. For each patient, all available archives including clinical data, hematoxylin and eosin (H&E)-stained slides and formalin-fixed paraffin embedded (FFPE) blocks were collected in this study. These patients were treated with surgical resection of primary gastric tumors between April 2010 and December 2015. Those diagnoses were confirmed by routine pathological examination after surgery. Ethics approval was obtained from the Ethics Committee of Xin Hua Hospital Affiliated to Shanghai Jiao Tong University School of Medicine. None of the patients received preoperative radiotherapy or chemotherapy. Pathologic parameters of all cases were reassessed according to the 4th edition of WHO classification for stomach tumors. The follow-up time was from initial diagnosis to September 2017 (range from 3 to 89 months).

### Data Collection

Refer to our data size, the clinical information and expression profiling of GSE62254 about 300 samples was downloaded from Gene Expression Omnibus (GEO) database and about 315 samples from TCGA (The Cancer Genome Atlas). The expression profiling of TCGA was downloaded from the UCSC Xena browser (http://xena.ucsc.edu/) and FPKM normalized. Their corresponding clinical information obtained from the online tool cbioportal (http://www.cbioportal.org/). All cases had determined subtypes but overall survival (OS) time of four cases and the AJCC pathological tumor stage of two cases were not available. Thus, 309 cases were analyzed for the clinicopathological characteristics and prognostic significance.

### MMR Immunochemistry (IHC) and EBV *in situ* Hybridization (ISH)

One representative FFPE block of the cancer region in each case was chosen for IHC and ISH analysis. Unstained 4-um thick tissue sections were tested by IHC antibodies to MLH1 (Clone M1, ready-to-use; Roche), PMS2 (Clone EPR3947, ready-to-use; Roche), MSH2 (Clone 219-1129, ready-to-use; Roche) and MSH6 (Clone 44, ready-to-use; Roche) for detection of MMR status, as well as chromogenic ISH with EBV-encoded RNA (EBER, Ventana) probe to prove EBV infection, using Benchmark automated staining device (Ventana Medical Systems, Roche, Switzerland) according to the manufacturer' instructions. All IHC and ISH stained sections were reviewed and scored independently by two professional digestive pathologists (WLF and WRF) without knowledge of previous clinical or pathological parameters.

The slides were evaluated as follows: at least one of the MMR proteins (MLH1, MSH2, MSH6, and PMS2) with complete loss of nuclear reactivity in tumor cells but consistently preserved nuclear staining in background non-tumor cells was taken as d-MMR(aberrant expression). When the tumor cells demonstrated intact nuclear immunostaining of all four MMR proteins, the tumor was judged as p-MMR (normal expression). For EBER, tumors with strong blue-black nuclear staining were considered positive.

### DNA Extraction and MSI Analysis

Total DNA was isolated from FFPE tumor and paired normal tissue samples though the DNA extraction kit (TIANGEN, Beijing, China) following the manufacturer's recommendation and was used for subsequent multiplex fluorescent PCR. MSI status was assessed with the amplification of six mononucleotide repeat markers (BAT25, BAT26, NR21, NR24, MONO27, and NR 27) described either in NCI (National Cancer Institute) - or Promega- panel. In addition, the final panel also contained one gender loci (Amel) and two pentanucleotide repeat markers (Peta C and Peta D) as internal controls. Co-amplification of these targets was performed on ABI 7500 using a 25 μl reaction volume advised by MSI-testing reagent kit (SINOMDgene, Beijing, China). The PCR conditions were carried out according to the operation protocols. Fluorescent PCR products were analyzed by capillary electrophoresis using an ABI 3500DX Genetic Analyzer (Applied Biosystems) and Genemaker software 2.0 (SINOMDgene, Beijing, China).

Tumors with instability at two or more of these 6 markers were defined as MSI-H, while those without instability or showing instability at only one marker were classified as MSS and Low- Microsatellite Instability(MSI-L) tumors, respectively.

### Construction of Weighted Gene Co-expression Network

To identify the key module that most associated with the OS time and status in 51 MSI cases and then investigate the underlying molecular connections, the weighted gene co-expression network analysis(WGCNA) was performed on the TCGA-STAD dataset (22). The variances of all genes were calculated and approximately top 6,000 genes were performed by use of the WGCNA R package.

To identify co-expressed genes, WGCNA use the soft thresholding power to determine the correlations between genes via the Sigmoid or Exponential function. In this study, the soft thresholding procedure was firstly performed to set the cutoff to identify the modules. Secondly, in order to identify the adjacent gene modules, the topological overlap dissimilarity measure (TOM) was used to calculate the correlation among genes. The hierarchical clustering was constructed and the minimum size was appropriately set to meet the different datasets' need. Thirdly, connecting modules to the external clinical traits could show us the key module that most associated with the OS time and status traits. After the key module had been identified, genes were put into the GO and KEGG enrichment analysis.

### Identification of the Hub Genes

After acquiring the module-trait relationships, the critical module was emerged. The pink module was obtained from the WGCNA that consisted of 250 nodes and 1,081 edges. This edge file was put into the Cytoscape software and constructed the gene co-expression network. The top five genes were *NUBP2, CTU1, ENDOG, SSNA1*, and *BCL7C* that the GS > 0.14 and MM > 0.75. But in the further validation in the GSE62254 dataset, the *CTU1* was not detected. Therefore, only four genes were considered the hub genes to be manifested and we selected the *NUBP2* and *ENDOG* as the typical hub genes to be in-depth functionally analyzed in TCGA-STAD dataset.

### Function Enrichment Analysis

After the key module was identified, genes were analyzed by The Kyoto Encyclopedia of Gene and Genomes (KEGG) and Gene ontology (GO). The clusterprofiler package in R software was used to realize these two gene enrichment analysis and the *P* < 0.050 (23).

### Gene Set Enrichment Analysis (GSEA) and Gene Set Variation Analysis (GSVA)

To probe the function of *NUBP2* and *ENDOG* in the dataset and elucidate their role in the good prognosis of MSI phenotype well, all MSI cases were divided into *NUBP2* or *ENDOG* high and low expression groups according to the median expression. The GSEA software downloaded from http://software.broadinstitute.org/gsea/ and annotated gene set c2.cp.kegg.v7.0.symbols.gmt. The top five significant pathways that derived from GSEA (*P* < 0.05) were shown in one graphic. GSVA was carried out in the high and low expression by the GSVA R package that also annotated gene set c2.cp.kegg.v7.0.symbols.gmt.

### Statistical Analysis

Clinicopathological parameters between groups were assessed for differences using the Pearson's X^2^ test, Yate's correction or Fisher's exact test. The Kaplan-Meier method (and the log-rank test) as well as Cox's proportional hazards regression model were used for univariate survival analysis. Multivariate survival analysis was performed by Cox's proportional hazards regression model. The performance of the model was evaluated by applying the area under curve of receiver operating characteristic (auROC). Overall survival (OS) was defined as the interval between diagnosis and date of death or last-documented contact with patient. The cut-off value of *NUBP2* and *ENDOG* was determined by the X-tile software (24). A two-sided *P*-value < 0.05 was regarded as statistically significant and all statistical calculations were done using STATA 10.1(stata corp., College Station, TX, USA) or R software(version 3.5.3).

## Results

### Prognosis and Potential Predictive Value of d-MMR/MSI-H Status in Different Cohorts

**Figure 1:**
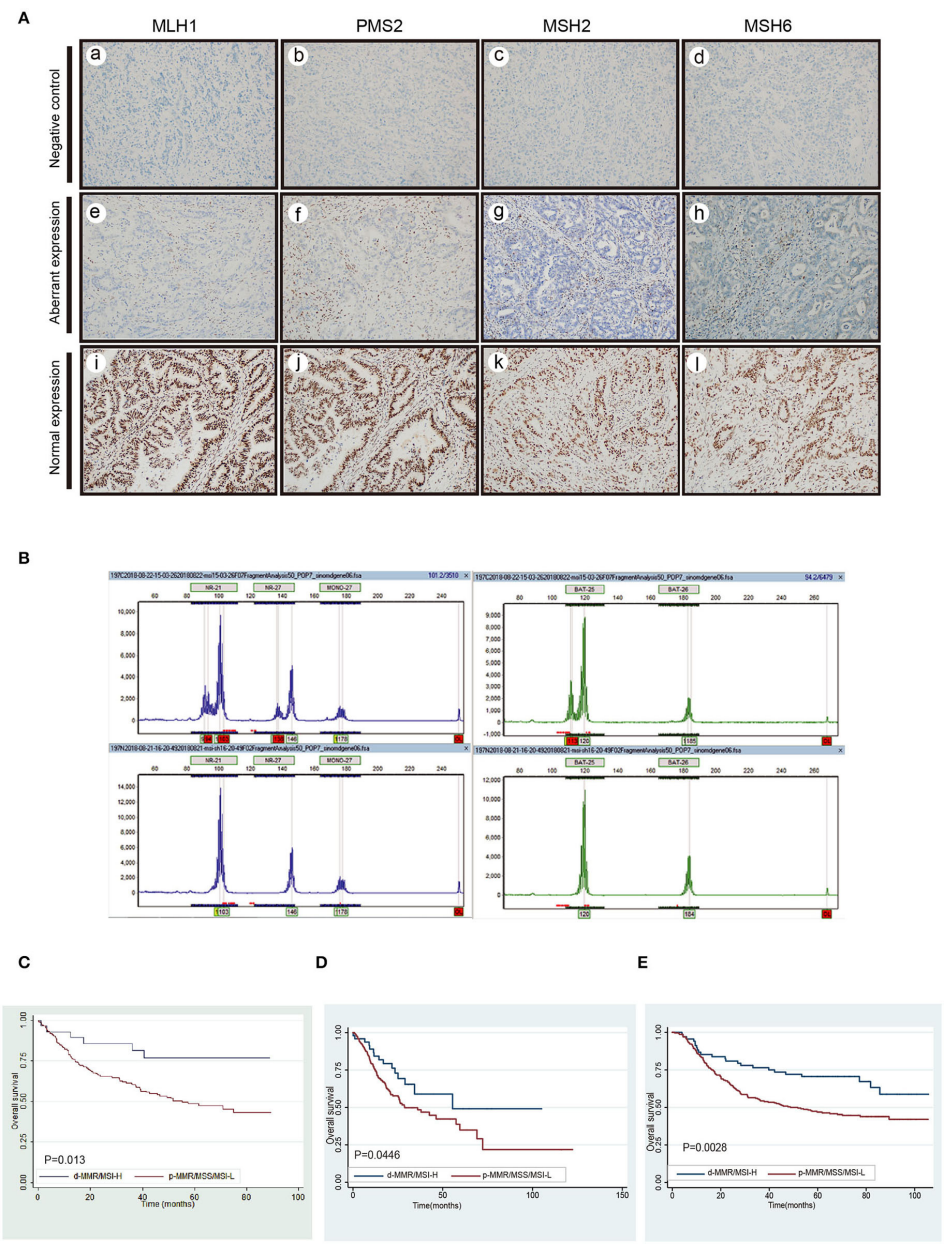
The detection of MMR, MSI and the survival analysis in different cohorts. **(A)** The negative control staining of MLH1 (a), PMS2 (b), MSH2 (c), and MSH6 (d). Complete loss of nuclear expression of MLH1 (e), PMS2 (f), MSH2 (g), and MSH6 (h) in tumor cells but preserved nuclear staining in background non-tumor cells(aberrant expression). The expressions of MLH1 (i), PMS2 (g), MSH2 (k), and MSH6 (l) in tumor cells are intact (normal expression) **(B)** The MSI-PCR testing results of MSI-H. **(C)** Survival analysis of d-MMR/MSI-H on the prognosis of gastric cancer in our study. **(D,E)** Survival analysis of MSI subtype in TCGA-STAD and GSE62254/ACRG cohort.

Of the 279 GC cases, the definite results of both IHC staining and MSI-testing were made in 275 cases. But four cases were detected to have the inconsistent results. Nuclear negative expression of MLH1, PMS2, MSH2, and MSH6 was seen in 27 (10.0%), 27 (10.0%), 1 (0.3%), and 1 (0.3%) in the rest of 271 cases, respectively. The normal and aberrant expression of MMR proteins were displayed in Figure 1A. MSI-PCR analysis revealed 28 cases of MSI-H, 2 MSI-L and 241 MSS. During this experiment, 27 cases showed instability at all six microsatellite loci and one case presented instability at five microsatellite loci except the Bat-6 (Figure 1B). Taken together, there were 28 cases with d-MMR/MSI-H and 243 cases with MMR-proficient/Low-Microsatellite Instability/Microsatellite stable (p-MMR/MSI-L/MSS) in 271cases. The detail was summarized in Table 1. Kaplan-Meier analysis and univariate analysis indicated that OS of GC patients with the d-MMR/MSI-H phenotype was better than that of patients with the p-MMR/MSI-L or MSS phenotype (*P* = 0.013) in the Figure 1C and Table 2. It was characterized by elderly age (*P* = 0.017), female (*P* = 0.014), without lymph node involvement (*P* < 0.0001), the lower depth of tumor invasion (*P* < 0.0001) and early TNM stage (*P* < 0.0001). We collected approximately 315 stomach adenocarcinoma from TCGA and described their clinical features as in the Table 3. Of the 315 cases, after six cases with undetermined subtype were removed, there were 50 MSI-H samples and the other 259 cases were considered as the MSI-low/MSS. The Kaplan-Meier survival analysis also indicated that patients with MSI phenotype had better prognosis than MSS among all different races which included Asian, White, Black or African American(*P* = 0.045) (Figure 1D). Then the GSE62254, derived from the ACRG research also illustrated the similar results. Among the 300 samples in dataset, 68 cases were MSI-H which also has a better correlation with prognosis (*P* = 0.003) (Figure 1E). The performance of the Cox's proportional hazards regression model was determined by applying the Receiver Operating Characteristic (ROC) curve analysis. The Area Under the Curve (AUC) value was 0.791. Therefore, this model has the predictive value for prognosis and was feasible.

**Table 1:** The relationship between EBV and d-MMR/MSI-H subtypes and clinicopathological parameters in 271 gastric cancers.

		**EBV**			**d-MMR/ MSI-H**		
		**(+)**	**(–)**	***P***	**(+)**	**(–)**	***P***
	**Median age**	**61**	**65**		**70.5**	**64**	
Age (%)	>65	4 (44.4)	122 (46.6)	1.000	19 (67.9)	107 (44)	**0.017**
	≦65	5 (55.6)	140 (53.4)		9 (32.1)	136 (56)	
Gender (%)	Male	6 (66.7)	176 (46.4)	1.000	13 (46.4)	169 (69.5)	**0.014**
	Female	3 (33.3)	86 (53.6)		15 (53.6)	74 (30.5)	
Location (%)	GEJ-cardia	2 (22.2)	27 (10.3)	0.248	1 (3.6)	28 (11.5)	0.197
	Non-GEJ-cardia	7 (77.8)	235 (89.7)		27 (96.4)	215 (88.5)	
Location (%)	Antrum	4 (44.4)	150 (57.3)	0.674	20 (71.4)	134 (55.1)	0.099
	Non- antrum	5 (55.6)	112 (42.7)		8 (28.6)	109 (44.9)	
Size (%)	<5	3 (33.3)	110 (28.6)	0.862	8 (28.6)	105 (43.2)	0.137
	≧5	6 (66.7)	152 (71.4)		20 (71.4)	138 (56.8)	
Differentiation (%)	Well-moderate	1 (11.1)	45 (10.7)	0.980	3 (10.7)	43 (17.7)	0.505
	Poor	8 (88.9)	217 (89.3)		25 (89.3)	200 (82.3)	
Lauren (%)	Intestinal	3 (33.3)	119 (45.4)	0.707	13 (46.4)	109 (44.9)	0.874
	Nonintestinal	6 (66.7)	143 (54.6)		15 (53.6)	134 (55.1)	
T (%)	T1-T3	8 (88.9)	180 (68.7)	0.355	26 (92.9)	162 (47.2)	**0.000**
	T4	1 (11.1)	82 (31.3)		2 (7.1)	81 (52.8)	
*N* (%)	N0	4 (44.4)	53 (20.2)	0.181	14 (50)	43 (17.7)	**0.000**
	N+	5 (55.6)	209 (79.8)		14 (50)	200 (82.3)	
M (%)	M0	8 (88.9)	259 (98.9)	0.127	28 (100)	239 (98.4)	1.000
	M1	1 (11.1)	3 (1.1)		0 (0)	4 (1.6)	
TNM (%)	I–II	5 (55.6)	97 (37)	0.436	19 (67.9)	83 (34.2)	**0.000**
	III–IV	4 (44.4)	165 (63)		9 (32.1)	160 (65.8)	
WHO (%)	Medullary	3 (33.3)	3 (1.1)	**0.000**	2 (7.1)	4 (1.6)	0.119
	Non-medullary	6 (66.7)	259 (98.9)		26 (92.9)	239 (98.4)	
WHO (%)	Papillary-tubular	3 (33.3)	143 (54.6)	0.359	19 (67.9)	128 (52.7)	0.127
	Non-papillary-tubular	6 (66.7)	119 (45.4)		9 (32.1)	115 (47.3)	

*The bold values indicate significant difference and P < 0.05*.

**Table 2:** Univariate and multivariable analysis of overall survival in 271 gastric cancer.

	**Univariate analysis**				**Multivariable analysis**		
	***P* (log-rank test)**	***P* (Cox's test)**	**HR**	**95% CI**	***P* (Cox's test)**	**HR**	**95% CI**
d-MMR/MSI-H status (Yes vs. No)	0.013	0.017	2.73	1.20–6.23	0.050	2.33	1.00–5.43
EBV (+ vs. –)	0.533	0.534	0.75	0.31–1.85			
Age	0.000	0.003	1.03	1.01–1.05	0.000	1.04	1.02–1.06
Sex (male vs. female)	0.092	0.093	1.39	0.95–2.95			
Location (antrum vs. nonantrum)	0.424	0.422	0.86	0.59–1.25			
Size (<5 vs. ≧5)	0.078	0.080	1.41	0.96–2.07			
Differentiation (well-moderate vs. poor)	0.226	0.228	1.39	0.82–2.35			
Lauren (intestinal vs. nonintestinal)	0.003	0.004	1.78	1.20–2.64	0.302	1.31	0.78–2.21
WHO (poorly cohesive components vs. remant)	0.000	0.000	2.04	1.40–2.97	0.130	1.48	0.89–2.46
pT stage (T1 + T2 vs. T3 + T4)	0.000	0.000	2.61	1.79–3.81	0.065	2.44	0.95–6.32
pN stage (N0 vs. N+)	0.002	0.003	2.35	1.34–4.12	0.360	0.65	0.26–1.63
M stage (M0 vs. M1)	0.467	0.476	0.49	0.07–3.51			
TNM (I + II vs. III + IV)	0.000	0.000	3.86	2.39–6.23	0.003	3.47	1.55–7.77

**Table 3:** The relationship between MSI subtype and clinicopathological parameters in 309 gastric cancers in TCGA.

	**d-MMR/MSI-H**			
		**(–)**	**(+)**	***P***
Number		259	50	
**Median age**				
Age (%)	≤ 65	124 (47.9)	14 (28.0)	0.015
	>65	135 (52.1)	36 (72.0)	
Gender (%)	Male	177 (68.3)	26 (52.0)	0.039
	Female	82 (31.7)	24 (48.0)	
Location (%)	Non-cardia	188 (72.6)	45 (90.0)	0.015
	Cardia	71 (27.4)	5 (10.0)	
Location (%)	Non-antrum	173 (66.8)	20 (40.0)	0.001
	Antrum	86 (33.2)	30 (60.0)	
Race (%)	NA	32 (12.4)	13 (26.0)	0.068
	White	165 (63.7)	25 (50.0)	
	Asian	52 (20.1)	11 (22.0)	
	Black	10 (3.9)	1 (2.0)	
T (%)	T1–T3	191 (73.7)	31 (62.0)	0.129
	T4	68 (26.3)	19 (38.0)	
N (%)	N0	70 (27.0)	19 (38.0)	0.217
	N+	185 (71.4)	31 (62.0)	
	NX	4 (1.5)	0 (0.0)	
M (%)	M0	227 (87.6)	46 (92.0)	0.564
	M1	18 (6.9)	3 (6.0)	
	MX	14 (5.4)	1 (2.0)	
Stage (%)	I–II	108 (41.7)	27 (54.0)	0.147
	III–IV	151 (58.3)	23 (46.0)	
NUBP2 (%)	Low	184 (71.0)	25 (50.0)	0.006
	High	75 (29.0)	25 (50.0)	
ENDOG (%)	Low	151 (58.3)	12 (24.0)	<0.001
	High	108 (41.7)	38 (76.0)	

### Clinical and Prognostic Features of EBV in Different Cohorts

**Figure 2:**
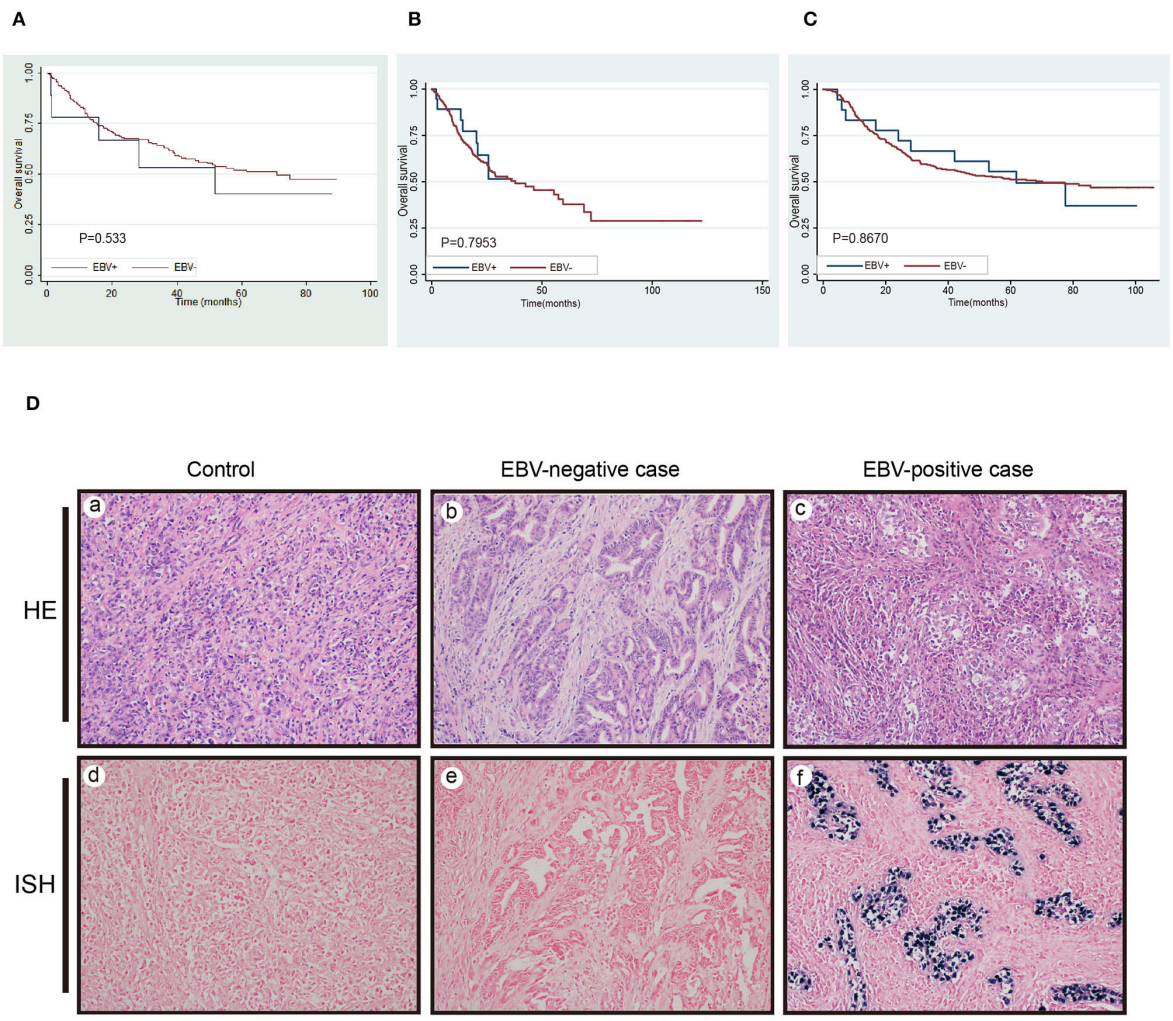
The detection of EBV infection and its survival analysis. **(A)** Survival analysis of EBV(+)gastric cancer in our study. **(B,C)** The survival analysis in TCGA-STAD and GSE62254/ACRG cohort for the same parameters. **(D)** The negative probe control of ISH in EBV+ infection cases (a) HE, and (d) ISH. One case with EBV-negativity (b) HE, and (e) ISH. One case with EBV-positivity (c) HE, and (f) ISH.

The incidence of EBV-positive GC in the 271 cases with consistent results was 3% (8/271). It has more frequent presence of EBV positive cases at GEJ/cardia-portion (*P* = 0.043) and medullary carcinoma (*P* < 0.0001) than EBV (–) cases (Table 1). Unlike d-MMR/MSI-H status, EBV infection itself by contrast was not prognostic factor in predicting OS of GC patients (*P* = 0.533, Figure 2A). In TCGA-STAD and GSE62254 dataset, 29 and 18 samples were detected to be EBV (+), respectively. The survival analysis also observed that it had no significant difference between EBV (–) and EBV(+) cases (*P* = 0.795, *P* = 0.867, Figures 2B,C). The EBER positive and negative case were displayed in Figure 2D.

### Identification of the Key Module That Associated With OS Time and Status and Its Annotation in MSI Sample

**Figure 3:**
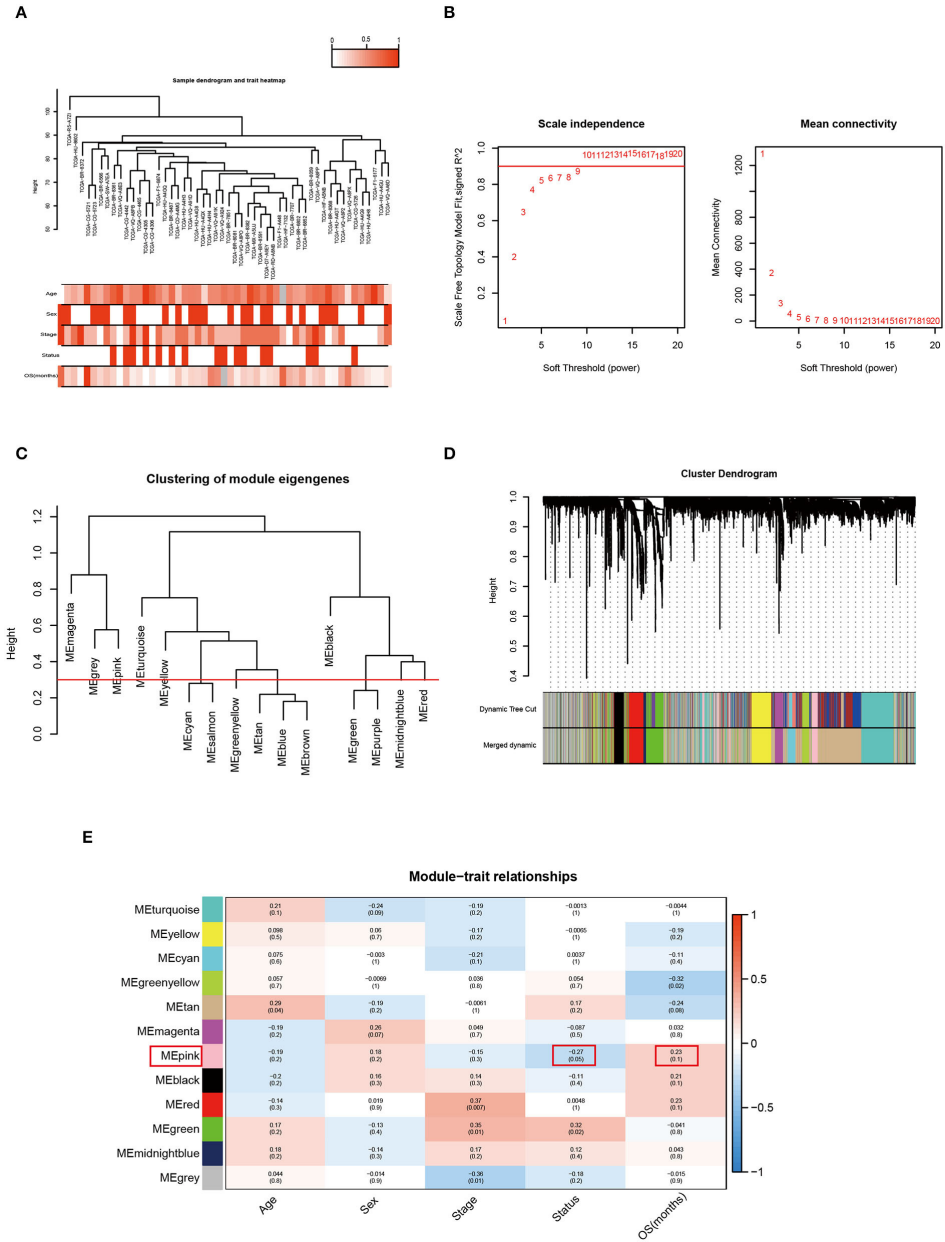
Weighted Correlation Network analysis was performed to construct the correlation between gene module and the clinical traits to find the key module that tightly associated with the prognosis. **(A)** Clustering the dendrogram of 51 MSI samples and the combination with its clinical traits. **(B)** Screening out the soft-thresholding power through scale independence and mean connectivity. **(C)** Clustering of the modules and set the criteria to merge the similar modules. **(D)** The dynamic cut tree after merging the similar modules. **(E)** The heatmap for module-trait relationships in DGC samples. The pink module was the key module with good status and long survival.

**Figure 4:**
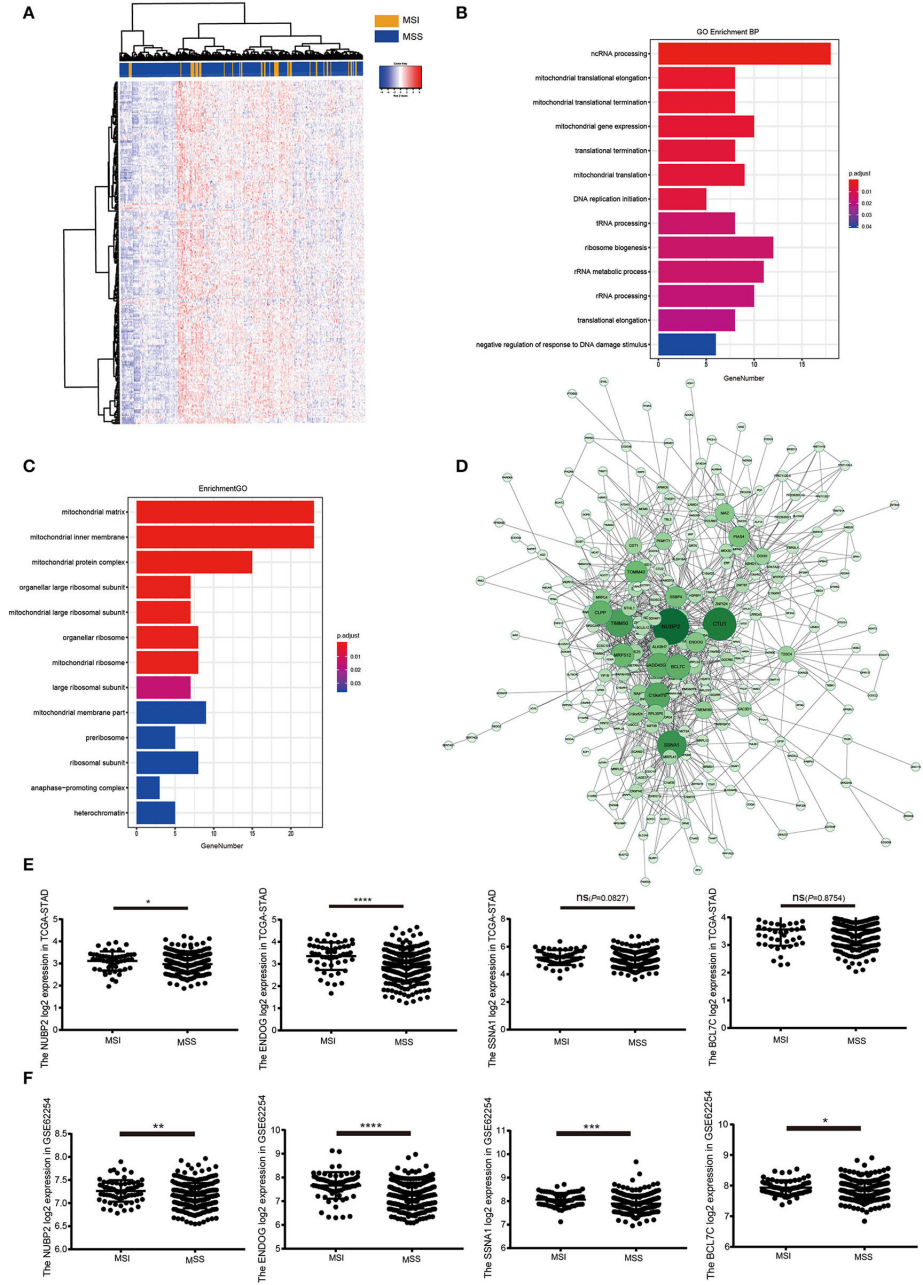
GO enrichment for the pink module and identification of the hub genes. **(A)** Heatmap for the expression pattern of all genes in pink module at MSI and MSS phenotype. **(B,C)** The biological process and cellular component for the pink module in GO annotations. **(D)** The coexpression network for the genes in pink module and identification of the hub genes. **(E)** The expression of *NUBP2, ENDOG, SSNA1* and *BCL7C* that had been log2 normalized in TCGA-STAD dataset in MSI (*N* = 51) and MSS group (*N* = 264). **(F)** The validation of *NUBP2, ENDOG, SSNA1* and *BCL7C* expression that had been log2 normalized in GSE62254/ACRG in MSI (*N* = 68) and MSS group (*N* = 232). *P < 0.05, **P < 0.01, ***P < 0.001, ****P < 0.0001.

In MSI subtype of GC, samples were clustered to detect the outliers while we did not delete any samples by average linkage method. The clinical trait data also could be input and the color representation of traits combined with the sample dendrogram (Figure 3A). The determination of soft-threshholding powers is the critical step to process this analysis. It was picked by the specific function in the WGCNA package and β = 9 was the most appropriate power to construct the adjacency (R^2^ = 0.870; Figure 3B). The ME dissimilarity threshold was set at 0.3 and twelve modules were manifested for this group (Figure 3C). Then we got the primary module separation and the dissimilarity of module eigengenes (ME) was calculated to merge the similar modules to form the merged dynamic tree (Figure 3D). Through connecting the gene module to clinical traits, pink module was highly negatively correlated with the status and also pink module had the longest survival. It represented these genes were most associated with good prognosis in the heatmap (Figure 3E). Then all genes were shown in the heatmap accoring to the MSI and MSS subtype in the pink module (Figure 4A). Meanwhile, the heatmap was also drawn for all genes in OS time and status (Supplementary Figures 2A,B). GO enrichment indicated that genes cluster to mitochondrial protein formation and ncRNA process (Figure 4B). These processes occurred in mitochondria and ncRNA may confer to the mitochondrial circle DNA (Figure 4C). There were lots of unknown molecular functions and GO enrichment could not be manifested by the clusterprofiler R package (Supplementary Figure 1B). Thus, the activity should be further detected by the hub genes to probe the alterations in the mitochondria.

### The Determination of the Hub Genes and Validation

The edge file, acquired from the WGCNA, put into the Cytoscape and genes were analyzed in the pink module (Figure 4D). According to the genes with high intra-modular connectivity ranked by the software, *NUBP2, CTU1, ENDOG, SSNA1*, and *BCL7C* could be considered as the hub genes. Also, these *CTU1, ENDOG, SSNA1*, and *BCL7C* had relative high GS and MM. The GS of *NUBP2* was relative lower but its MM was such high that could not be neglected. As they were selected from the MSI samples in TCGA-STAD, hub genes were validated by the GSE62254/ACRG dataset. In GSE62254 dataset, *CTU1* could not be observed and only the other four hub genes were used to be further analyzed to assist us to uncover the specific activity that tightly associated with the good prognosis in MSI samples. *NUBP2* and *ENDOG* had significant difference between MSI and MSS subtype in TCGA-STAD. But the expression of *BCL7C* and *SSNA1* had no significant difference in these two subtypes (Figure 4E). There were 68 MSI samples in GSE62254. Others were considered the MSS phenotype. Then we found that the four hub genes highly expressed in MSI samples and had more significant difference than that was in MSS in GSE62254 (Figure 4F). Then all samples were divided into high and low expression group according to the appropriate cutoff value of these hub genes. The expression of *NUBP2* (*P* = 0.006) and *ENDOG* (*P* < 0.001) had significant difference between MSI and MSS subtypes (Table 3).

### GSEA and GSVA for the Hub Genes

**Figure 5:**
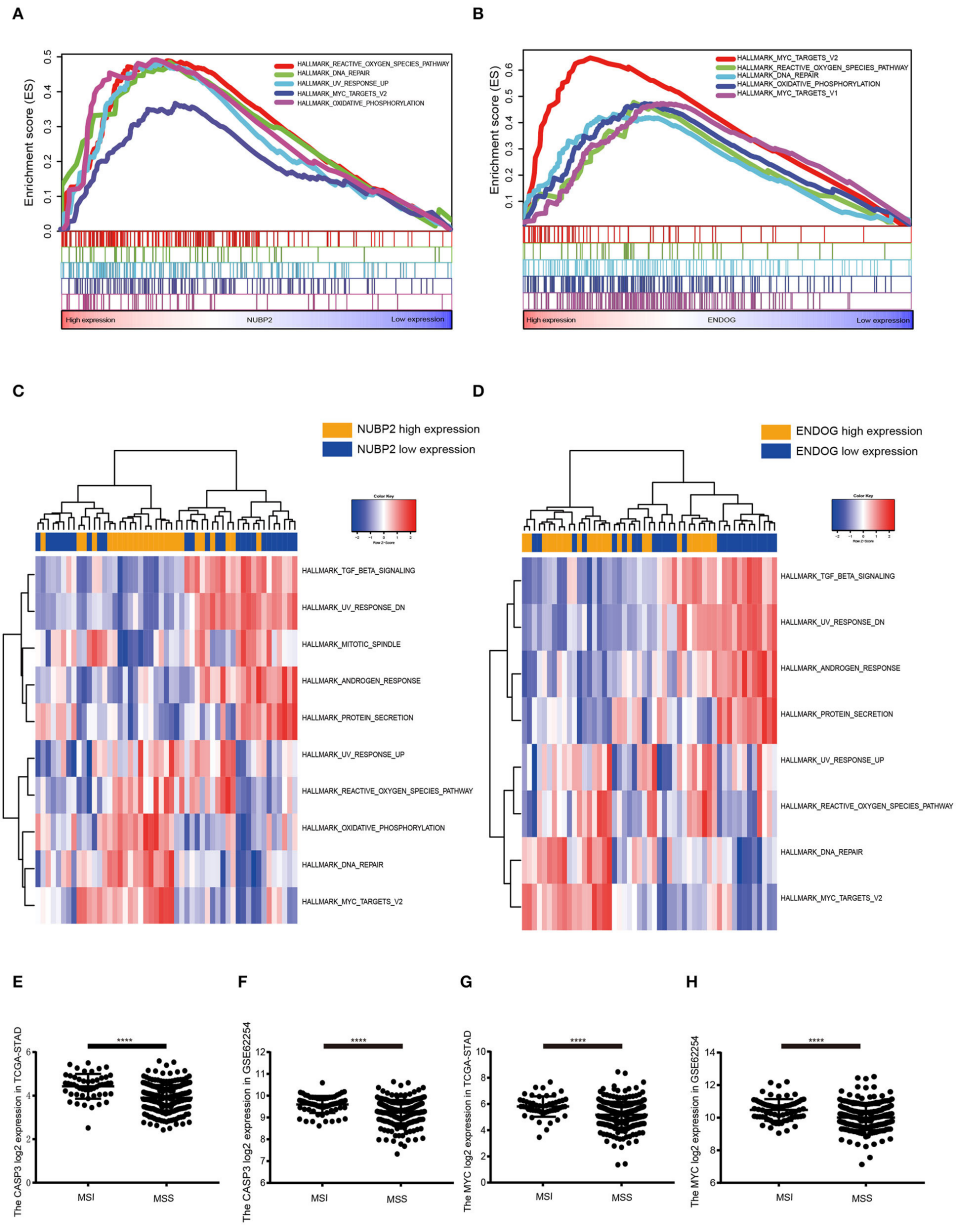
Gene set enrichment analysis (GSEA) and gene set variation analysis(GSVA) for the *NUBP2* and *ENDOG* in TCGA-STAD. **(A,B)** The top five gene sets(according to the enrichment score) enriched in the high expression of single hub gene for HALLMARK gene sets. **(A)** *NUBP2*; **(B)** *ENDOG*. **(C,D)** The heatmaps of differentially expressed pathways for single hub gene through the calculation of GSVA. **(E,F)** The log2 normalized expression of *MYC* and *CASP3* in MSI and MSS samples in TCGA-STAD dataset. **(G,H)** The log2 normalized expression of *MYC* and *CASP3* in GSE62254 dataset. ****P < 0.0001.

GSEA and GSVA were conducted to further shed light on the function of hub genes by comparing the differential expression group. According to the median expression of *NUBP2, CTU1, ENDOG, SSNA1*, and *BCL7C*, all cases were divided into the high and low expression group. Based on the nominal *P* < 0.050 and the normalized enrichment score (NES), top five KEGG pathways were illustrated in *ENDOG* and *NUBP2* highly expressed group (Supplementary Figures 3A,B). It more inclined to enrich in oxidative phosphorylation, glutathione metabolism and DNA repair. The common HALLMARK gene sets were reactive oxygen species pathway, oxidative phosphorylation, MYC targets and DNA repair that characterized by the mitochondrial impairment and oxidant stress (Figures 5A,B). The GSVA for *NUBP2* and *ENDOG* made similar conclusions as well (Figures 5C,D and Supplementary Figures 3C,D). *CTU1, BCL7C*, and *SSNA1* were carried out the same analysis and shown in Supplementary Figures 4, 5. It could be concluded that both the cell impairment and anti-impairment associated pathways existed in this group. Nevertheless, the expression of *MYC* and *CASP3*, encoding the caspase3, was higher in MSI than it was in MSS samples in TCGA-STAD and GSE62254 (Figures 5E–H). Therefore, oxidative phosphorylation and reactive oxygen species pathways facilitate the apoptosis and had significant difference between MSI and MSS subtype. In addition, we performed the GSEA in GSE62254/ACRG in which the samples derived from Samsung Medical Center (Asian ethnicity) for status and the differential expression of hub genes (Supplementary Figures 6A–C). The results were similar with that performed in TCGA-STAD dataset and consistent with the above investigations as well (Supplementary Figure 6D).

## Discussion

The immune checkpoint therapy has become the most dazzling star in recent years since it has revealed the therapeutic efficacy in melanoma (25). The ever-increasingly comprehensive research also enhanced it to be the hotpoint in different cancer types. Nevertheless, not all types of cancer could actually benefit from the immunotherapy while not all patients had response for the determined effective cancer (26). Under this circumstance, the identification and well understanding of the subtypes greatly assisted to improve the efficacy of the anti-PD1 therapy. The recent proposal of TCGA molecular classification broadened our view of GC molecular characteristics by highlighting four main subtypes that summarized the western population. Then the Asian Cancer Research Group (ACRG) also came up with a molecular classification by analyzing the expression profiling of Asian population. Both of these two classifications involved the MSI and EBV phenotype. Investigations had confirmed that immune checkpoint blockade gained the better efficacy in MSI and EBV associated GC (27–30). The discovery of correlations between this classification and differential therapeutic response reaffirmed that clinical-relevance of each subtype was caused by distinct molecular mechanism of GC. Nonetheless, the clinical course of immunotherapy related EBV (+) or MSI-H GC is not fully understood. As far as we concerned, current data that concentrate on the associations of EBV infection and MSI phenotype with clinical parameters and outcome of GC was relatively scarce in East-Asia. To address it, our retrospectively analysis of the EBV infection and MSI status were chosen based on reliable detection methods in a cohort of Chinese GC patients (*n* = 279).

Currently, PCR combined with capillary electrophoresis and IHC was routinely performed to detect the MSI (31, 32). In this study, an integrated testing panel containing the mononucleotides of BAT-25, BAT-26, NR21, NR24 and MONO27 were carried out in these 279 cases. Meanwhile, IHC was also adopted to test the four MMR associated proteins MLH1, MSH2, MSH6, and PMS2. Studies often focused on the consistency rate of these two methods varied a lot (91.2–97.8%) (33, 34). Previously, investigations indicated the proportion of d-MMR/MSI-H was ~8.2–44.5% in different cohort while the incidence was 10.3% (28/271) in our study. It reconfirmed that D-MMR/MSI-H GC was related to older age, female, lower depth of tumor invasion, without frequency of lymph node metastasis and lower TNM stage, but was not consistent with tumor size, distal location, medullary carcinoma and intestinal subtype that previously reported. Even so, there was still a trend toward higher rates of antrum-located location, large size (≧ 5), medullary carcinoma and papillary-tubular type seen in our d-MMR/MSI-H GC. Scientists reported that MSI-H was significantly related with higher survival at 15 years of follow-up and an independent prognostic factor that reminded us its predictive role relied on further prolonged follow-up. Besides that, we collected the expression profiling of GC tissues and corresponding clinical traits from TCGA (*n* = 315) and GEO (GSE62254/ACRG). The cohort consisted of Asian, Black or African American, Native Hawaiian or other Pacific island and white in the TCGA-STAD dataset. In 2014, TCGA did not investigate the significant OS differences between MSI and other subtypes. But it had a better prognosis in this TCGA dataset which was downloaded by us. This was reconfirmed in Asian population involved in the GSE62254/ACRG by Kaplan-Meier analysis.

At present, EBV infection could be tested by several methods, such as polymerase chain reaction (PCR), electron microscopy, southern blot hybridization, IHC and ISH that was considered as the gold-standard test. Due to limited sample size (3%), EBVaGC was just associated with proximal location and medullary carcinoma, but not with reported characteristics of male predominance in this study. Additionally, consistent with some previous research, it was also found that this subtype could not reflect a long-term survival (12). It made the identical conclusion from the TCGA-STAD and GSE62254. During this process, we have also observed that these two situations (EBV positive and d-MMR/MSI-H status) are virtually mutually exclusive in line with previous reports though both of these subtypes showed a good response to immunotherapy (2, 3, 10, 12, 21). Obviously, the distinct PD-L1 associated expression profile owed by them need to be further studied.

The different prognostic influence between EBV and MSI subtypes was worthful to be explored as their common efficacy to immune checkpoint blockade. Nevertheless, the MSS subtype could respond to the immunotherapy with EBV infection and there were little cases with both MSI and EBV phenotype (30). It means that at least two distinct molecular mechanisms exist in these two subsets and the immunotherapy can be controlled based on this. Some recent investigations have reported the clinical characteristics for the status of EBV infection in GC. There was approximately average 10% EBV positive cases in GC samples worldwide (35, 36). For Latvia GC population, EBV positivity was a favorable prognostic factor in GC while it had no significant difference in other relative large cohort (37). Not only the intrinsic alterations of MSI and EBV infected cells but also the tumor microenvironment (TME) contributes to these clinical characteristics to some extent. On the one hand, the metabolic differences manifested that lipid metabolism was evident in MSI tissues as the fatty acid synthase (FASN) increased in colorectal cancer is associated with MSI (38, 39). Sirt1, the critical histone deacetylase that cross the mitochondrial metabolism and DNA damage repair, correlated with MSI (40). Meanwhile, some fatty acid biosynthesis related enzymes FASN and PLA2G4A decreased in EBVaGC and could lead to the worse survival. Similarly, EBV infection could make the metabolic reprogramming and it is the foundation of the poor clinical prognosis in GC patients (41). On the other hand, the discrepancy of tumor microenvironment was another controversial topic and may interpret the molecular mechanism. Recently, bioinformatics analysis come up with the TMEscore and Immunoscore which also could be consider as a prognostic and predictive tool for GC by a large scale microarray data (42–44). During the process of accessing the tumor purity, higher TMEscore was associated with a good prognosis and characterized by the response to virus and IFNγ that was consistent with the features of MSI in latest researches (42). Furthermore, the activation of immune response commonly observed in MSI and EBV subtypes and immunomicroenvironment appears complicated and play a role in metabolic reprogramming as well. Of note, T cell metabolism could not be easily ignored as it involved in the IFN-γ and fatty acid synthesis in the TME (45).

As the good prognosis was such evident for MSI, it was appealing to explore the critical factors that associated with the longer survival. The WGCNA provide the pink module that could be considered as the key one with the better status and longest overall survival in MSI samples. Genes predominantly enriched in mitochondria or ribosome and played a role in the process of ncRNA, mitochondrial translation elongation or termination and mitochondrial gene expression. As amount of unknown molecular functions in these modules, the specific function of this module was probed by the hub genes to detect the concrete activity to ensure the OS for MSI cases. Apparently, not only the mitochondria associated proteins but also the genes involved the MSI conditions. Mitochondrial activities need to be in-depth studied and may uncover the origins of MSI.

The fetched five hub genes by WGCNA, *NUBP2, CTU1, ENDOG, SSNA1*, and *BCL7C* were illustrated by Cytoscape. They were revalidated by the GSE62254/ACRG and had more significant difference in MSI cases than MSS. Then *NUBP2* and *ENDOG* was the top two genes close to the centrality could reflect the activity of this module in mitochondria to largely extent. Oxidative phosphorylation, reactive oxygen species pathway, MYC targets, glutathione metabolism and DNA repair were the obvious pathways that tightly associated with the high expression of hub genes and the alteration of mitochondrial translation. On the basis of these parts, we could conclude that the better prognosis associated with the function of mitochondrial proteins that mainly played a great part in oxidant phosphorylation, ROS pathway and MYC targets as the apoptosis was increasing. Actually, the glutathione metabolism, base excision repair and DNA repair reflected the activity of antioxidant response and anti-impairment. But weigh the two factors, high expression of apoptosis associated gene determined that the injury factors play a dominant role in MSI subtype.

Until now, rare investigations reported the relationships between the MSI phenotype and mitochondrial activity. Mitochondrial microsatellites instability (mtMSI) was easier to be ignored than the nuclear MSI. As the alterations emerged in mitochondrial matrix according to the GO annotation, the ncRNA process and DNA replication initiation had a great probability to represent the mitochondrial DNA variations. Considerable investigations had revealed its links with the prognosis of colorectal cancer while rare researches involved in GC (46). Though the function of mitochondrial DNA is less powerful than the nuclear DNA, it is convenient to regulate the oxidative phosphorylation system (OXPHOS) (47). *NUBP2*, the nucleotide binding protein 2, encodes adenosine triphosphate (ATP) and metal-binding protein that modulate the iron-metabolism that was essential for ATP production and mitochondrial metabolism (48). It was the essential component that could assemble the iron-sulfur clusters through the process of cytosolic iron-sulfur cluster assembly (CIA) outside of the mitochondria (49, 50). Compared with the hypoxic microenvironment, the upregulation of *NUBP2* indicated the normoxia and ensure the oxidative phosphorylation in the MSI samples in GC. The normal activity of oxidative phosphorylation decreased the tumor cell atypia and its malignancy that lead to the better prognosis. *ENDOG*, the Endonuclease G, was the nuclear encoded gene and its corresponding protein mainly localized in mitochondria. This protein is capable of initiating the mitochondrial DNA replication by generating the RNA primers (51, 52). On the one hand, it was the downstream effector of caspase-3 and facilitated the Myc-induced genetic instability and apoptosis (53, 54). On the other hand, *ENDOG* regulate the mRNA alternative splicing of hTERT (52, 55). As the non-active splice variant hTERT increased, the activity of telomerase is suppressed and lead to the short telomere which acquired the replicated senescence for tumor cells (56). On the basis of these reasons, tumor cells have more opportunities and prone to be induced apoptosis and cell senescence in MSI subtype.

## Conclusions

Taken together, we classified the clinical characteristics of MSI and EBV in Chinese GC cohort to some extent with the limited cases. Combining with the public datasets, we summarized that MSI could serve as a prognostic factor for good survival while it had no significant difference in EBV associated cases. The prognostic value tightly associated with the oxidative phosphorylation system, reactive oxygen species and MYC targets pathways through the modulation of mitochondria. The glutathione metabolism and DNA repair were also active but the antioxidant response could not resist the accumulation of ROS and genetic instability that contribute to more opportunities for cell apoptosis in MSI samples. Based on these discoveries, some attractive strategies of up regulating the *ENDOG* or *NUBP2* could be utilized to increase the oxidative phoshorylation for MSS subtype which could imitate the easily apoptotic effects. Certainly, more experimental and clinical trials should apply to optimize and achieve the potential to acquire the similar prognostic effects like MSI in other subtypes.

## Data Availability Statement

Publicly available datasets were analyzed in this study, these can be found in The Cancer Genome Atlas via the UCSC Xena browser (http://xena.ucsc.edu/); the NCBI Gene Expression Omnibus (GSE62254).

## Ethics Statement

The studies involving human participants were reviewed and approved by Ethics Committee of Xin Hua Hospital Affiliated to Shanghai Jiao Tong University School of Medicine. The patients/participants provided their written informed consent to participate in this study. Written informed consent was obtained from the individual(s) for the publication of any potentially identifiable images or data included in this article.

## Author Contributions

LC and YS performed the research study, analyzed the data, and wrote the paper. KW, WG, JY, and JL supported during performing of the experiments, collected patient data, and contributed essential reagents and laboratory equipment. RW and LW conceived and designed the study. All authors contributed to the article and approved the submitted version.

## Conflict of Interest

The authors declare that the research was conducted in the absence of any commercial or financial relationships that could be construed as a potential conflict of interest.

## Abbreviations

GC: Gastric Cancer
MSI: Microsatellite Instability
TCGA: The Cancer Genome Atlas
EBV: Epstein-Barr virus
EBVaGC: Epstein-Barr virus associated gastric cancer
TCGA-STAD: Stomach adenocarcinoma samples in The Cancer Genome Atlas
GEO: Gene Expression Omnibus
ACRG: Asian Cancer Research Group
MSS: Microsatellite Stable
WGCNA: Weighted Gene Co-expression Network Analysis
ROS: Reactive Oxygen Species
MMR: Mismatch Repair
d-MMR/MSI-H: Mismatch Repair deficiency/High-Microsatellite Instability
p-MMR/MSI-L: MMR-proficient/Low-Microsatellite Instability
KEGG: The Kyoto Encyclopedia of Gene and Genomes
GO: Gene ontology
GSEA: Gene Set Enrichment Analysis
GSVA: Gene Set Variation Analysis
OXPHOS: Oxidative Phosphorylation System
FPKM: Fragments Per Kilobase per Million.
