# Supplementary figures and images for: The Better Survival of MSI Subtype Is Associated With the Oxidative Stress Related Pathways in Gastric Cancer
Source: Front Oncol. 2020 Jul 28;10:1269. doi: 10.3389/fonc.2020.01269 (PMC7399340; doi:10.3389/fonc.2020.01269)

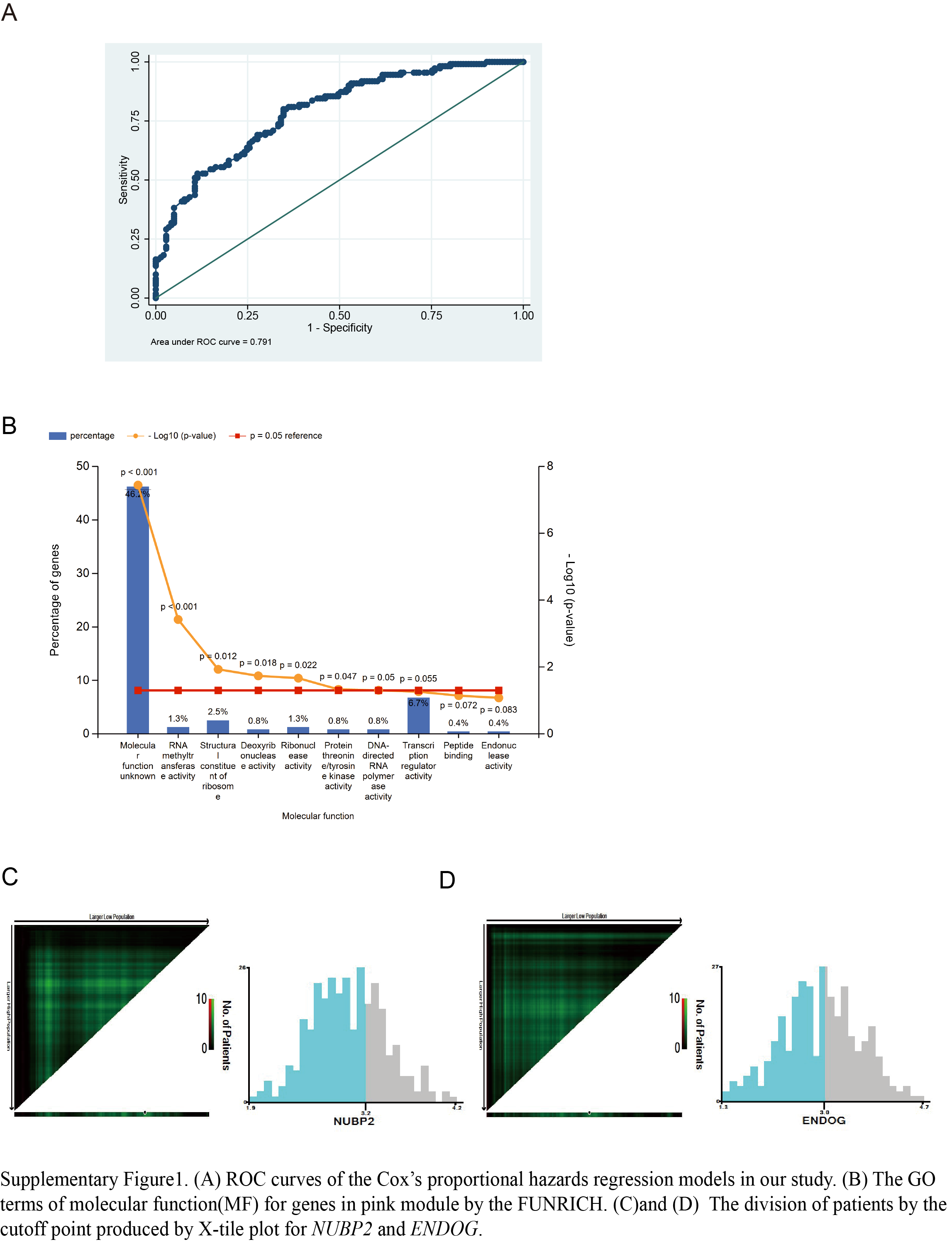

Supplement: Supplementary file 1 [file Image_1.TIF]

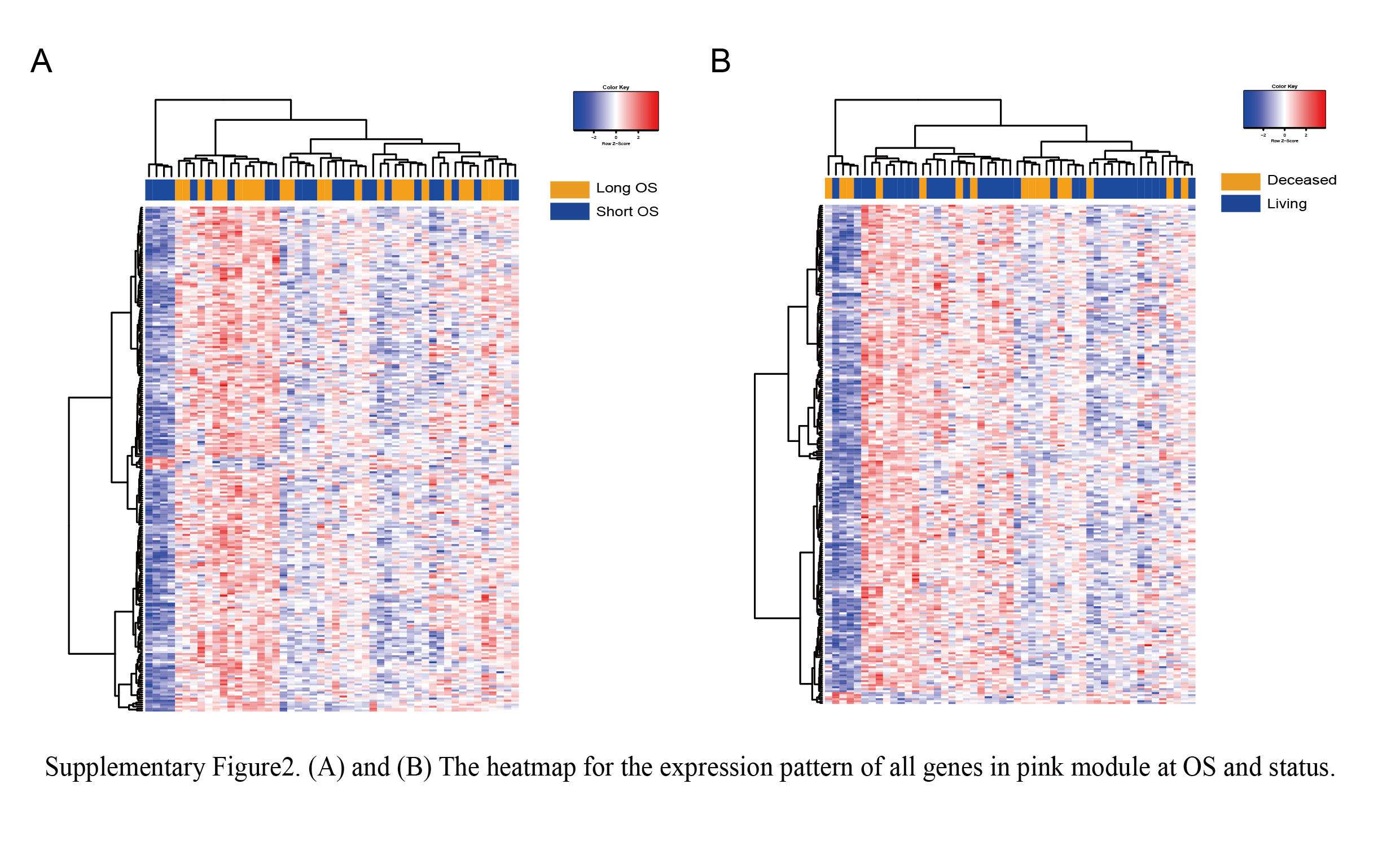

Supplement: Supplementary file 2 [file Image_2.TIF]

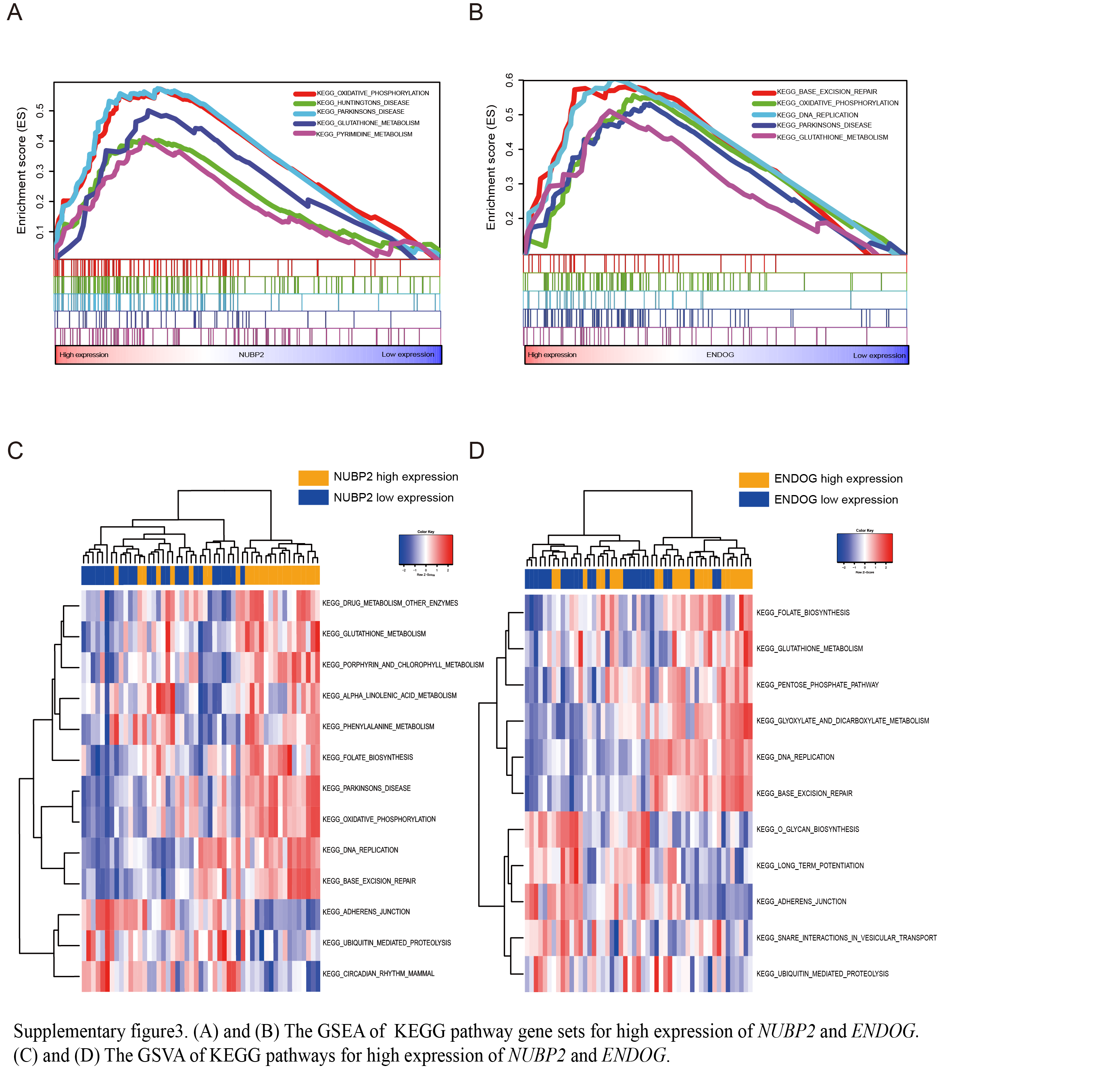

Supplement: Supplementary file 3 [file Image_3.TIF]

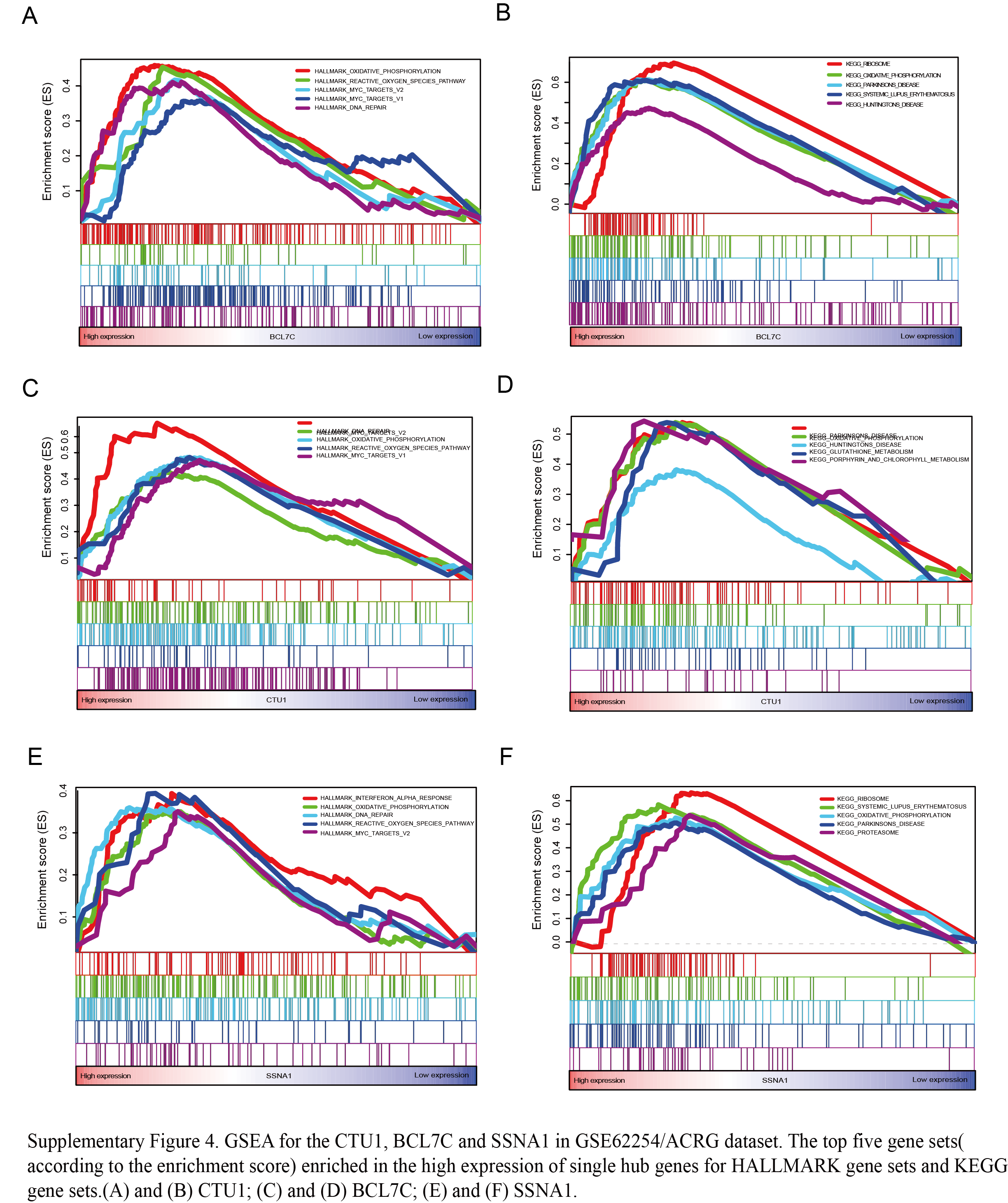

Supplement: Supplementary file 4 [file Image_4.TIF]

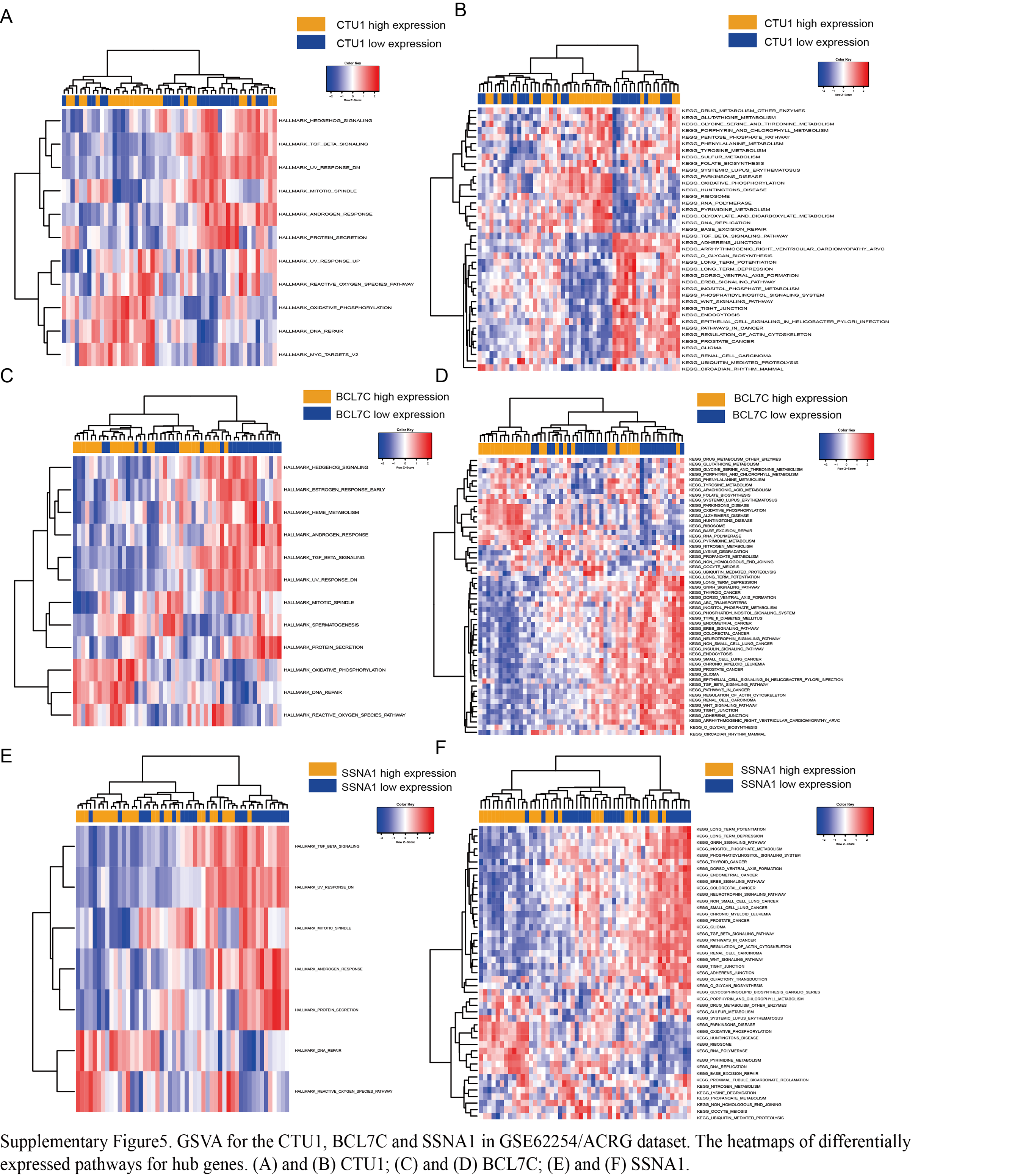

Supplement: Supplementary file 5 [file Image_5.TIF]

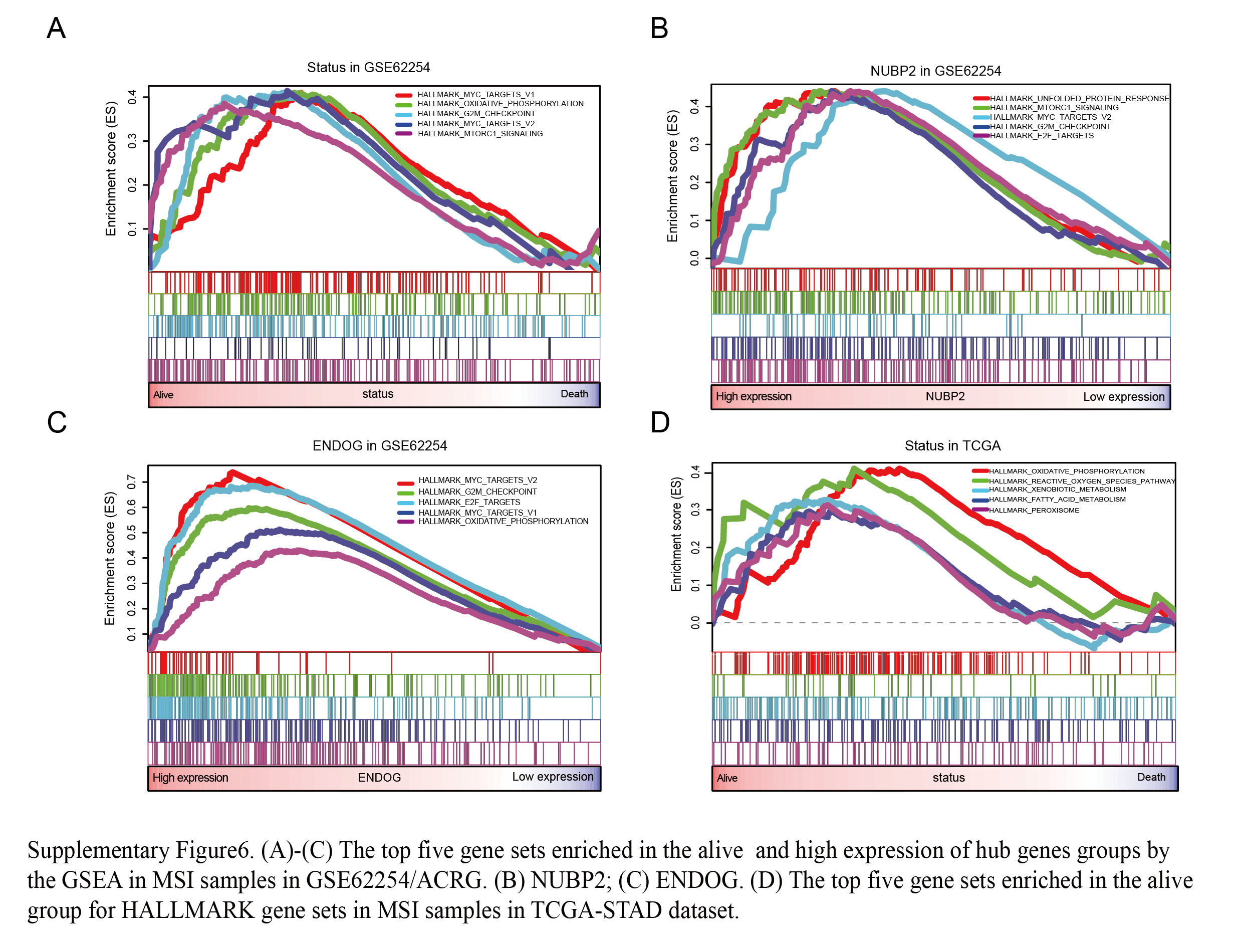

Supplement: Supplementary file 6 [file Image_6.TIF]
